# Flavor assessment of a lactic fermented vinegar described in Japanese books from the Edo period (1603–1867)

**DOI:** 10.1016/j.heliyon.2024.e32344

**Published:** 2024-06-04

**Authors:** Naoyuki Yanagihara, Maeda Mayumi, Jun Yoshikawa, Sayuri Akuzawa, Akira Fujii, Masanobu Nagano, Yukimichi Koizumi, Kenji Maehashi

**Affiliations:** a Yanagihara Cooking School of Traditional Japanese Cuisine, 1-7-4 Akasaka, Minato-ku, Tokyo 107-0052, Japan; b Department of Fermentation Science and Technology, Graduate School of Tokyo University of Agriculture, 1-1-1 Sakuragaoka, Setagaya-ku, Tokyo, 156-8502, Japan; c Faculty of Applied Biosciences, Tokyo University of Agriculture, 1-1-1 Sakuragaoka, Setagaya-ku, Tokyo, 156-8502, Japan; d Sakamoto Kurozu Inc., 21-15, Uenosono-cho, Kagoshima, 890-0052, Japan

**Keywords:** Edo period, Japanese cuisine, Kurozu, Lactic fermentation, Rice vinegar, Sourness, Umami

## Abstract

**Aims:**

Rice vinegar is a traditional fermented seasoning in Japan, and its production remained unchanged for over 800 years until the Edo period. However, based on the available information regarding rice vinegar production methods from this period and the results of reproduction experiments, we speculated that unlike the modern-day acetic fermented vinegar, rice vinegar produced during the Edo period was lactic fermented. *Main methods:* To verify this assumption, we analyzed the flavor components of Honcho**,** a lactic fermented product prepared using a method described in books, including "*Honchoshokkan*" from the Edo period, by capillary electrophoresis/time-of-flight mass spectrometry, high-performance liquid chromatography, gas chromatography mass spectrometry, and taste sensor analysis. Sensory evaluation was also conducted to assess validation as a seasoning.

**Results:**

Honcho contains 2 % lactic acid, which gives it its acidity, and small amounts of other nonvolatile acids, but significantly lower levels of acetic acid (0.188 ± 0.015 g/100 mL, *p* < 0.01). It contains more than double the free amino acids of Kurozu, a modern rice vinegar, and more glutamic acid. Boiling to remove ethanol from yeast fermentation concentrated the free amino acids 1.5 times. Sensor taste analysis showed Honcho had weaker acidity but stronger umami taste than commercial rice vinegar. The volatile compounds related to acetic acid fermentation were significantly different between Honcho and Kurozu. Boiling increased Honcho's acidity, mainly through non-volatile acids.

**Significance:**

These findings provide evidence to indicate that Honcho was an acidic seasoning for heat-cooking, which is uncommon in Japanese cuisine today and is mentioned in Edo period books. This seasoning contains many amino acids, implying that it adds umami flavor, not only the sourness of modern vinegar.

## Highlights

• Lactic fermented vinegar is a sour seasoning composed mainly of 2 % lactic acid.
• This vinegar is rich in free amino acids, especially glutamic acid.
• Uses for sourness and umami in heat-cooked dishes.
• Lactic fermented rice vinegar may have been used as a seasoning in Edo Japan.

## Graphical abstract

**Figure:**
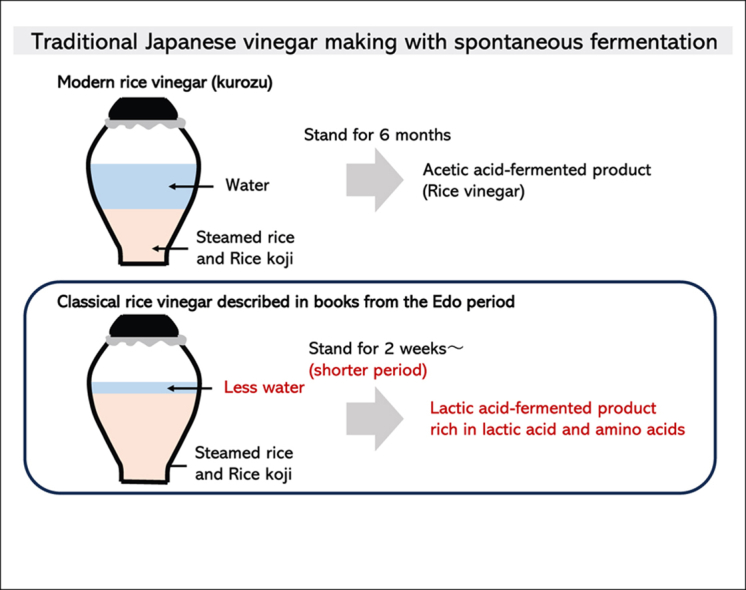

## 1 Introduction

Rice vinegar is a traditional fermented seasoning, along with soy sauce, miso, mirin, and sake, that are indispensable component of Japanese cuisine. In recent years, various biological functions of rice vinegar have been identified, including antihypertensive [1] and antithrombotic effects [2], and obesity reduction [3], and their health effects have attracted attention [4,5].

In Japan, rice vinegar is industrially produced through two-stage fermentation, including alcohol fermentation, followed by acetic fermentation using steamed rice, rice koji, and water as ingredients, and yeast and acetic acid bacteria as seed cultures [6]. In modern Japan, there are two main methods for rice vinegar production: traditional static surface fermentation and modern submerged fermentation [7]. In static fermentation, which is generally employed in traditional vinegar production, sake is first produced by alcohol fermentation, with a crepe pellicle of acetic acid bacteria floating on the surface of the sake, and ethanol is converted to acetic acid after approximately one month of acetic fermentation [6]. Although the manufacturing methods and functionalities of rice vinegar have been investigated in detail, little is known about the history of its manufacturing methods in Japan. It is believed that the vinegar production method was introduced from China to southern Osaka in Japan in the 4th century, and spread nation-wide, forming the basis of traditional Japanese vinegar production methods [8,9]. The earliest description of vinegar production in Japan can be found in the *'Engishiki’* book, which was written in 927. The production method hardly ever changed for more than 800 years, probably until vinegar manufacturing companies were founded in the middle of the Edo period (1603–1867), based on the descriptions of 15 books published between 927 and 1866 [10]. However, the charged water ratio was very low in these methods compared with the present age [10]. In addition, the fermentation period was as short as one week to one month, mostly during the summer, which is considerably different from the present day [10]. It is likely that after the middle of the Edo period, modern two-stage fermentation became a common method of rice vinegar production. However, in limited areas of Japan, rice vinegar is still produced in pottery jars using a spontaneous fermentation method, similar to the traditional method, until the Edo period. However, in this method the charged water ratio is approximately four times the total rice, and the fermentation period is half a year, starting in spring or autumn [10]. This modern method produces an amber rice vinegar called Kurozu [4].

In our previous study, we concluded that the rice vinegar production method practiced in Japan until the Edo period was different from modern methods based on the characteristics of the 33 vinegar manufacturing methods described in books from the Edo period [10]. We reproduced the rice vinegar production methods with different water/material ratios described in four Edo period books, including *Honchoshokkan* (1697), to determine what vinegar can be made. Methods with low water/material ratios, as described in books from the Edo period, produce fermented products containing lactic acid as the main acid without acetification [10].

In this study, we used “Honcho” to refer to the lactic fermented product of the rice vinegar production method described in *Honchoshokkan*. We propose that lactic fermented products, such as Honcho, were produced as rice vinegars and used for cooking in Japan until the Edo period. To test this hypothesis, it is necessary to know whether lactic fermented products can act as seasoning products. In this study, we determined the chemical composition of Honcho and evaluated its seasoning characteristics.

## 2 Materials and methods

### 2.1 Materials

Four types of Japanese classical rice vinegars and one modern rice vinegar were manufactured at Sakamoto Kurozu Inc. (Kagoshima, Japan), which has been preparing Kurozu using traditional methods since the beginning of the 19th century. Since vinegar has been spontaneously produced at this location for over 200 years, the microbial environment for vinegar production is well adapted and consistent. Four types of rice vinegars, Honcho, Bankin, Wakan, and Kokubyaku were manufactured using the methods described in books from the Edo period, *Honchoshokkan* (1697), *Bankinsugiwaibukuro* (1732), *Wakansansaizue* (1712), and *Kokubyakuseimisyu* (1798). The manufacturing method has been previously described [10]. Briefly, steamed rice, rice koji, and water were placed in this order in a pottery pot (diameter 42 cm, caliber 14 cm, height 60 cm, content 54 L), as indicated in Table 1, and stirred gently with a stick. A piece of charcoal and iron nails were placed on top of the ingredients, and a paper lid and pottery lid were placed on the pot. The reason charcoal and iron nails were placed on top of the ingredients remains unknown and is not provided in the books. Raw rice was polished to 1–2%, and rice koji made by Sakamoto Kurozu Inc. was used. Four pots of each vinegar were prepared, spontaneously fermented without the addition of any microbes, and aged outdoors for 195 (first year) or 199 days (second year). As a control, a modern rice vinegar, Kurozu was manufactured using the same procedure as Honcho, except for the ratio of ingredients and lack of charcoal and iron nails, which we named “Sakamoto” in this study. The water/rice ratios (v/v) of the rice vinegars are shown in Table 1. Commercially available Kurozu (Sakamoto Kurozu Inc.) and rice vinegar (manufactured in Japan) were also used. Rice vinegars were produced twice over two years. In the spring of the first year, Honcho, Kokubyaku, and Sakamoto were manufactured, and in the spring of the second year, Honcho, Bankin, Wakan, and Sakamoto were manufactured. In this study, Honcho, Bankin, Wakan, and Sakamoto manufactured in the second year and Kokubyaku manufactured in the first year were used for the analysis. Four lots of Honcho were manufactured, and consequently, two high lactic acid products (Honcho 1) and two low lactic acid products (Honcho 2) were obtained, as described previously [10].

**Table 1:** Components used in rice vinegar production.

Rice vinegars	Steamed rice (kg)	Rice koji (kg)	Water (L)	Total (kg)	Water/rice ratios (v/w)^a^
Honcho	15.1	6.7	24.2	46.0	1.5
Bankin	12.4	2.8	30.9	46.1	2.7
Kokubyaku	14.2	6.32	25.2	45.7	1.7
Wakan	7.8	3.5	34.7	46.0	4.1
Sakamoto	8.1	3.7	35.0	46.8	4.0

a Water/rice ratio was calculated based on the total raw rice weight, which was calculated as (steamed rice + rice koji)/1.35.

Commercially available rice vinegar (rice vinegar) was used for amino acid analysis, taste analysis and sensory evaluation. Additionally, commercially available Kurozu (Kurozu) was used for amino acid analysis and acidity experiments. Both of these were used as controls for Honcho.

### 2.2 Organic acid analysis

The organic acids in the vinegars were measured by high-performance liquid chromatography (HPLC) using a Shodex OA system (Showa Denko, Tokyo, Japan) equipped with a Shodex Rspak KC-811 column (i.d. 8.0 × 300 mm, Showa Denko) and a Shodex Rspak KC-G guard column (i.d. 6.0 × 50 mm) with 3 mM perchloric acid as a mobile phase at a flow rate of 1.0 mL/min. The eluate was reacted with a reaction solution (0.2 mM bromothymol blue/15 mM disodium phosphate, flow rate of 1.0 mL/min) at 60 °C and detected at OD_445nm_. The samples subjected to HPLC included Honcho 1 and 2, Bankin, Wakan, and Sakamoto (aged for 199 days), as well as Kokubyaku (aged for 195 days).

### 2.3 Free amino acid analysis

Free amino acids in vinegar were measured by IDEA Consultants, Inc. (Osaka, Japan). The analysis was performed using an OPA/FMOC pre-labeled HPLC on an Agilent 1100 System equipped with an Agilent Poroshell 120 EC-C18 column (i.d. 3.0 mm × 150 mm, 2.7 μm). The column temperature was 40 °C, and the flow rate was 0.5 mL/min. Elution was performed with a linear gradient of mobile phase A (5 mM sodium phosphate buffer, pH 7.6) and mobile phase B (methanol:acetonitrile:water = 45:45:10). A fluorescence detector was used for detection, with an excitation wavelength of 340 nm and an emission wavelength of 450 nm for OPA, an excitation wavelength of 266 nm, and an emission wavelength of 305 nm for FMOC.

Honcho 1 (aged 199 days), Kurozu, and rice vinegar were used as the samples in this analysis. Honcho 1 (boiled) was obtained by boiling to remove large amount of alcohol for approximately 2 min. Subsequently, using an alcohol densitometer (Alcomate AL-3, Woodson, Tokyo, Japan), 2.25 % w/v alcohol was detected.

### 2.4 Metabolome analysis by CE/TOFMS

Metabolome analyses of Honcho and Sakamoto, both aged 199 days, were conducted by Human Metabolome Technologies (HMT) Co., Ltd. (Yamagata, Japan). The samples were analyzed in the cation and anion modes of a capillary electrophoresis/time-of-flight mass spectrometer (CE/TOFMS) using an Agilent CE-TOFMS system (Agilent Technologies Japan, Ltd., Tokyo, Japan) with a fused silica capillary (i.d. 50 μm × 80 cm). The mass spectrometer was operated in positive (ESI positive) and negative (ESI negative) ion modes under the following conditions: MS capillary voltage, 4000 V and 3500 V; MS scan range, mass to charge ratio (*m*/*z*) of 50–1,000, Sheath liquid, and HMT Sheath Liquid. For peaks detected by CE-TOFMS, the automatic integration software MasterHands ver.2.17.1.11 was used to automatically extract peaks with an s/n of 3 or more to obtain *m*/*z*, peak area values, and migration times (MT). The obtained peak area values were converted into relative peak area values. The detected peaks were collated and searched for all substances registered in the HMT metabolite library and Known-Unknown library of HMT Co., Ltd., based on the *m*/*z* and MT values. The metabolome analysis was conducted for one lot each of Honcho 1, Honcho 2, and Sakamoto. Honcho 1 and Honcho 2 were made using the same fermentation process but were found to have different lactic acid bacterial flora. Further, Honcho 1 and Honcho 2 were dominated by *Lactobacillus* sp. and *Pediococcus pentosaceus*, respectively [10].

### 2.5 Analysis of aromatic components

The aromatic compounds in the vinegar samples were analyzed using the headspace method with a GCMS-TQ8040 NX trap system (Shimadzu Corporation, Kyoto, Japan). The column used was DB-WAX (i.d. 0.25 mm × 60 m, 0.25 μm, Agilent Technologies Japan, Tokyo, Japan), and amyl acetate was used as the internal standard material. Samples (100 μL) saturated with sodium chloride were placed in TORAST HS vials (Shimadzu GLC, Tokyo, Japan) and the temperature program was executed by agitating the vials at 50 °C for 30 min. The column was held at 50 °C for 5 min, increased to 250 °C at a rate of 10 °C/min, and then maintained at 250 °C for 10 min. The mass spectrometry range was set between 40 and 400 *m*/*z* in scan mode.

### 2.6 pH and acidity determination

The pH was measured using a pH meter. Acidity was calculated as w/v% acetic acid from the amount of NaOH 0.1 mol/L (0.1 N) solution required to neutralize the vinegar sample to pH 8.3.

### 2.7 Taste analysis

Taste analysis was conducted at the Q'Sai Analysis Laboratory (Fukuoka, Japan) using a taste recognition device, TS-5000Z (Intelligent Sensor Technology, Japan), with five sensors: BT0 for sourness, C00 for bitter-complexity and bitterness, AE1 for astringent-stimulus and astringent, CT0 for saltiness, and AAE for umami and umami-koku. Vinegar samples were assessed after 3-fold dilution with distilled water, and the interpolated addition value was obtained first. We confirmed whether the estimated taste calculated from the potential difference was actually perceived by performing a taste presence/absence judgment standard and sensory test of the device. Next, the measured value of rice vinegar, compared with the interpolation addition value, was considered to be 0.00, and the interpolation difference value was calculated. Each experiment was performed in triplicate.

### 2.8 Sensory evaluation

The taste of Honcho and dishes seasoned with Honcho was evaluated by 19 professional Japanese, Western, and Chinese chefs using rice vinegar as a control. Samples were prepared as follows: 1. vinegar itself; 2. irisu; 3. su-miso; 4. sushi-meshi; and 5. chicken suiri. Sample 2 was prepared by mixing 45 mL vinegar, 15 mL sake, and 3 g salt, heated to boil and then cooled. Sample 3 was prepared by mixing 60 g white rice miso, 3 g sugar, and 30 mL vinegar, followed by gentle heating with stirring until the mixture became shiny. Sample 4 was prepared by cooking 333 g rice in 460 mL water, then adding 1.5 g salt dissolved in 40 mL vinegar, and then cooling it with air. For sample 5, 50 g chicken fillet cut into small pieces was sprinkled with a small amount of salt, baked, added to 15 mL vinegar, and heated gently until the moisture evaporated. For taste, a five-grade visual analog scale was used to measure the sourness intensity, sourness stimulus, and umami intensity. In detail, each end of a 10 cm line segment signified “weak” and “strong,” and panelists were asked to check the position corresponding to the sensory intensity they felt for each item. The length was considered the sensory intensity.

### 2.9 Statistical analysis

Experimental taste analysis and sensory evaluation data were assessed using analysis of variance with Excel Statistics version 4.04 software (Social Survey Research Information Co., Ltd., Tokyo, Japan). One-way analysis of variance was performed, followed by a two-tailed test to assess the difference in the mean values between vinegar samples using Tukey's method with a significance level set at 5 %.

### 2.10 Limitations of the study

The reproduction of the rice vinegar production method from the Edo period is not feasible without the cooperation of the company that has been manufacturing vinegar since that period. We conducted experiments on the reproduction of vinegars twice over two years using the company's facilities, materials, and pottery jars with the cooperation of Sakamoto Kurozu Co. Ltd. However, it has not been confirmed whether similar products can be obtained at other vinegar manufacturing companies. In this study, a detailed component analysis and sensory test were conducted only on Honcho, which was representative of lactic fermentation products manufactured according to the books of the Edo period in a previous study.

### 2.11 Ethics approval

Sensory evaluation was performed with the approval of the Ethics Committee of the Tokyo University of Agriculture on Experiments and Surveys on Human Subjects (approval number 2114). The study complies with all regulations and confirmation that informed written consent was obtained.

## 3 Results and discussion

### 3.1 Comparison of organic acids in Honcho and Sakamoto

We compared the flavor components of Honcho, a rice-fermented product manufactured according to Edo-period books, and Sakamoto, a rice vinegar produced using modern manufacturing methods. While acetic acid bacteria were detected in Sakamoto, no growth of acetic acid bacteria was observed in Honcho, where lactic acid bacteria were dominant [10].

The four types of products obtained using different water/material ratios, as shown in Table 1, were compared with that of Sakamoto. Sakamoto is a modern vinegar with a water/rice ratio of 4, while Honcho has a much lower water/rice ratio of 1.5. The water/rice ratio is approximately the same between Honcho and Kokubyaku, Sakamoto and Wakan, whereas the water/rice ratio of Bankin falls in between that of Honcho and Sakamoto. As shown in Table 2, the amount of lactic acid measured by HPLC using an ion exclusion chromatography column was approximately seven- and three-fold higher in Honcho 1 and Honcho 2, respectively, than that in Sakamoto. Kokubyaku, manufactured using a similar method as Honcho and Bankin, but with a water/rice ratio between Honcho and Sakamoto (Table 1), contained higher amounts of lactic acid than Sakamoto. In our previous study, we showed that Honcho produced 2780 mg/100 mL of lactic acid [10]. In contrast, the acetic acid content in the Honcho lots was much lower than that in Sakamoto. Lactic acid is the main component in Honcho and other classical rice vinegars, followed by ethanol, with the exception of Wakan. Our previous study showed that the ethanol content was 12 % for Honcho and Kokubyaku, 9 % for Bankin, and 0.1–0.2 % for Wakan and Sakamoto in our previous study [10]. Therefore, lactic acid is considered a characteristic taste of classical rice vinegar [10]. Wakan, prepared using a method similar to Sakamoto, contained lower amounts of lactic acid and higher amounts of acetic acid than Sakamoto. However, the original method described in *Wakansansaizue* indicated that the fermentation period was 14 days and lactic acid fermentation peaked at 1 %, but not acetic acid fermentation [10]. Acetic acid was only detected at a concentration of 0.188 ± 0.015 g/100 mL in Honcho (mean of four lots of Honcho 1 and Honcho 2) was at a significantly low level compared with Sakamoto (*p* < 0.01). Lactic acid is consumed by acetic acid bacteria; thus, we speculate that all rice vinegar production methods described in the Edo period books produced lactic fermented products with 1–2 % lactic acid.

**Table 2:** Organic acid quantification using HPLC or CE/TOFMS in Honcho, Sakamoto, and other rice vinegars manufactured following descriptions in Edo period books.

Organic acids	Content (mg/100 mL)					
	Honcho		Bankin	Kokubyaku	Wakan	Sakamoto
	1	2				
Lactic *^1^	2314*^2^	1077*^2^	1076 ± 142*^4^	2550*^2^	110.6 ± 11.2*^3^	326.2 ± 6.0*^3^
Lactic	2318	1004	990	–	–	294
Citric	0.98	7.62	ND	–	–	ND
Gluconic	31.51	56.53	0.66	–	–	4.55
Isocitric	1.37	0.65	0.65	–	–	0.75
Malic	1.11	0.54	0.54	–	–	0.71
Pyruvic	0.86	5.23	ND	–	–	ND
Succinic	61.63	57.36	47.14	–	–	24.65
Acetic *^1^	203.5*^2^	167.1*^2^	137.1 ± 23.0*^4^	270*^2^	7053 ± 191*^3^	6950 ± 137*^3^

*1 determined using HPLC.

*2 average of each 2 lots.

*3 mean ± SD (n = 3).

*4 mean ± SD (n = 4), ND and − indicate not detected and not determined, respectively. Organic acids other than lactic acid and acetic acid were determined using CE/TOFMS in metabolome analysis (n = 1).

Metabolome analyses using CE/TOFMS were used to compare other non-volatile organic acids in the mash supernatants from Honcho, Bankin, Kokubyaku, Wakan, and Sakamoto. The lactic acid content measured by metabolome analysis was consistent with that measured by HPLC. The results of Sakamoto (Table 2) are consistent with those of Koizumi et al. [11], who showed that most Kurozu products contain the highest amount of lactic acid among the non-volatile acids. The content of all non-volatile acids other than lactic acid was also higher in Honcho than in Sakamoto, especially gluconic acid and succinic acid. Some acetic acid bacteria are known to assimilate lactic, malic, tartaric, citric, malonic, succinic, and fumaric acids [12]. Therefore, we considered that these organic acids remained in Honcho because the acetic acid bacteria did not assimilate them. In addition to lactic acid, succinic acid, which is known for its umami taste, is relatively abundant in sake and plays an important role in its taste [13]. The acidic tastes of lactic and succinic acids in sake increase significantly with warming, resulting in a smooth and comfortable sensation [13]. Because succinic acid is almost twice as abundant in Honcho as in Sakamoto, it may affect the umami and acidic tastes of Honcho. Succinate has been identified as a substrate for intestinal gluconeogenesis and improves glucose homeostasis [14]. The gluconic acid content was also higher in Honcho than in Sakamoto. Gluconic acid has a mild taste that refreshes the acidity [15]. Gluconic acid increases intestinal microbiota and has attracted attention because of its health benefits [16]; therefore, it is expected to contribute to the taste and health benefits of Honcho.

### 3.2 Free amino acid content

The free amino acid content in Honcho, boiled Honcho, rice vinegar, and Kurozu was examined using HPLC (Table 3). Honcho or boiled Honcho were compared with two modern vinegars, rice vinegar and Kurozu, to assess its suitability as a seasoning. All free amino acid contents were higher in Honcho than in rice vinegar and Kurozu, which had similar to the metabolome analysis results to Honcho and Sakamoto (data not shown). The total amino acid content of Kurozu in this study was twice as high as that reported by Koizumi et al. [17], but nearly 10 times higher than the content of rice vinegar. Furthermore, total amino acid content of Honcho was twice as high as that of Kurozu in this study. The total amount of amino acids increased by 1.5-fold in boiled Honcho, compared with Honcho, however, boiling did not alter its amino acid composition.

**Table 3:** Free amino acids in Honcho, rice vinegar, and Kurozu.

Amino acids	Content (mg/100 mL) and composition (%)*^2^				
	Rice vinegar*^1^	Kurozu*^1^	Honcho 1	Honcho 1 (boiled)*^3^	Average of 11 Kurozu products*^4^
Asp	4.9 (8.4)	22.0 (4.4)	110.0 (9.2)	170.0	8.5 ± 8.1 (4.1)
Glu	5.1 (8.8)	30.0 (5.9)	130.0 (10.9)	200.0	15.8 ± 12.3 (7.7)
Asn	1.3 (2.2)	0.5 (0.1)	22.0 (1.8)	33.0	N/A
Ser	2.9 (5.0)	32.0 (6.3)	79.0 (6.6)	120.0	11.5 ± 4.7 (5.6)
Gln	ND	ND	0.2 (0.0)	0.5	N/A
His	0.8 (1.3)	2.8 (0.6)	38.0 (3.2)	58.0	4.7 ± 2.5 (2.3)
Gly	0.3 (0.5)	41.0 (8.1)	79.0 (6.6)	120.0	12.6 ± 5.6 (6.1)
Thr	1.6 (2.7)	28.0 (5.5)	57.0 (4.8)	86.0	9.6 ± 5.0 (4.7)
Arg	7.8 (13.4)	3.4 (0.7)	24.0 (2.0)	37.0	6.2 ± 6.2 (3.0)
Ala	5 (8.8)	99 (19.6)	160 (13.4)	240	35.8 ± 21.5 (17.4)
Tyr	3.3 (5.7)	4.8 (1.0)	35.0 (2.9)	54.0	6.9 ± 4.9 (3.3)
Val	3.1 (5.3)	53.0 (10.5)	110.0 (9.2)	160.0	18.0 ± 8.4 (8.7)
Met	0.7 (1.1)	12.0 (2.4)	28.0 (2.3)	43.0	4.7 ± 2.0 (2.3)
Trp	0.2 (0.4)	0.8 (0.2)	0.4 (0.0)	0.4	N/A
Phe	3.2 (5.5)	17.0 (3.4)	14.0 (1.2)	22.0	11.3 ± 6.2 (5.5)
Ile	2.5 (4.3)	42.0 (8.3)	75.0 (6.3)	110.0	11.8 ± 5.6 (5.7)
Leu	5.9 (10.1)	60.0 (11.9)	120.0 (10.0)	180.0	22.1 ± 9.0 (10.8)
Lys	3.1 (5.3)	32.0 (6.3)	53.0 (4.4)	81.0	17.4 ± 14.0 (8.4)
Pro	6.5 (11.2)	25.0 (4.9)	61.0 (5.1)	95.0	10.7 ± 5.1 (5.2)
Total	58 (100)	505 (100)	1196 (100)	1810	205.8 ± 82.3 (100)

*1 Commercially available products.

*2 Composition is shown in parentheses.

*3 Honcho was boiled to remove alcohol.

*4 Data is represented as mean ± SD of 11 kurozu products in Koizumi et al. (1987). ND and N/A indicate not detected and not available, respectively.

Rice vinegar contained a high proportion of aspartic acid, glutamic acid, arginine, leucine, and proline; Kurozu contained a high proportion of alanine, valine, and leucine; and Honcho contained a high proportion of aspartic acid and glutamic acid in addition to alanine, valine, and leucine. High proportions of aspartic acid and glutamic acids were common in rice vinegar and Honcho. Generally, rice vinegar has a high amino acid content [18]. In particular, pot-made Kurozu contains more amino acids than regular rice vinegar [17]. In comparison, Honcho contained 20 times more free amino acids than rice vinegar did, and twice as many free amino acids as Kurozu. Honcho exhibited a remarkably high glutamic acid content of 130 mg/100 mL. Moreover, we observed a similarly high glutamic acid content (129.28 ± 22.06, n = 6) in spontaneously fermented Honcho using the same koji in our laboratory (data not shown). Reports have shown that some traditional Chinese vinegars, such as Shanxi-aged vinegar, have approximately 10 times more free amino acids than Japanese rice vinegar [11]. Differences in production methods may lead to significant variations in the amino acid content. Honcho is a seasoning with a particularly high umami component compared to modern rice vinegar.

### 3.3 Other metabolites

We performed metabolome analysis using TOF/MS in CE-TOFMS cation and anion modes on substances registered in the Human Metabolome Technologies (HMT) metabolite library, known-unknown peak library, and HMT peptide list (2–4 residues). Honcho, made with much lower water/rice ratio, is representative vinegar in the Edo period. As its chemical composition are unknown, its minor chemical composition was compared with that of Sakamoto, which is a modern vinegar. A total of 412 peaks (326 cations and 86 anions) were detected, and 305 compounds were identified from the *m*/*z* and MT values of substances registered in the HMT metabolite library. In addition, based on the *m*/*z* values of the HMT library peptide, 107 unidentified peaks were assigned to candidate peptide names. Table 4 lists 47 candidate compounds detected in Honcho and/or Sakamoto with over 1.0E-03 of relative peak area with considerable (more than double) differences between both samples in metabolomic analysis of 305 identified compounds. This list excludes amino acids and organic acids. Almost all 47 compounds listed in Table 4 were also detected in another lot of Honcho and Bankin (data not shown); therefore, they are considered to be common compounds found in rice vinegar.

**Table 4:** Candidate compounds detected in Honcho and/or Sakamoto.

HMT DB	*m/z*	MT	Relative peak area			Ratio^a^
Candidate compound name			Honcho 1	Honsho 2	Sakamoto	Honcho 1/Sakamoto
Glucose 6-phosphate	259.024	10.32	1.7E-03	1.3E-04	ND	1<
Kojic acid	143.035	20.89	2.1E-03	4.2E-04	ND	1<
Dyphylline	255.108	20.93	1.2E-02	1.6E-02	3.8E-04	33
Mevalonic acid	147.068	8.97	1.2E-03	9.6E-04	1.1E-04	11
5-Aminovaleric acid	118.087	7.47	9.0E-02	1.5E-02	9.3E-03	9.6
2-Hydroxybutyric acid	103.040	10.28	3.5E-03	3.5E-03	4.5E-04	7.7
5-Oxoproline	128.036	10.07	1.7E-01	1.4E-01	2.8E-02	6.0
Threonic acid	135.031	9.75	2.1E-03	1.8E-03	3.8E-04	5.6
*O*-Succinylhomoserine	218.068	12.87	1.3E-03	1.0E-03	2.5E-04	5.4
4-Acetamidobutanoic acid	144.067	8.85	1.4E-03	9.3E-04	2.6E-04	5.2
3-Aminopropane-1,2-diol	92.071	6.75	1.3E-03	2.1E-03	2.8E-04	4.5
β-Ala	90.055	6.90	4.7E-03	5.3E-03	1.2E-03	3.8
Glyceric acid	105.020	10.99	1.3E-03	3.1E-03	3.6E-04	3.7
(Gly, Pro)	173.093	8.46	1.3E-02	1.7E-02	3.7E-03	3.6
2-Isopropylmalic acid	175.061	14.55	1.9E-03	1.9E-03	5.7E-04	3.4
Glucuronic acid or Galacturonic acid	193.036	8.41	5.9E-03	5.5E-03	2.2E-03	2.7
Agmatine	131.129	4.86	2.0E-01	2.4E-01	7.7E-02	2.6
Acetylcholine	146.118	7.15	4.5E-02	5.1E-02	1.8E-02	2.5
*N*-Acetylalanine	130.052	9.32	1.7E-03	1.7E-03	6.9E-04	2.4
2-Phenylethylamine	122.097	7.28	4.8E-02	4.4E-02	2.0E-02	2.4
Ethanolamine	62.061	5.98	1.7E-02	1.8E-02	7.2E-03	2.4
*meso*-Tartaric acid	149.010	23.64	2.6E-03	2.4E-03	1.1E-03	2.3
Methionine sulfoxide	166.054	11.18	5.0E-03	5.8E-03	2.1E-03	2.3
Stachydrine	144.102	10.83	5.6E-02	5.8E-02	2.7E-02	2.1
Cadaverine	103.123	4.69	4.7E-02	5.1E-02	2.3E-02	2.0
Choline	104.107	6.44	1.5E-01	1.4E-01	7.6E-02	2.0
Betaine	118.087	10.61	7.8E-02	7.9E-02	3.9E-02	2.0
Cytosine	112.051	6.83	7.9E-03	2.4E-03	4.0E-03	2.0
Trigonelline	138.056	9.83	1.6E-03	2.0E-03	8.0E-04	2.0
Histamine	112.088	4.50	5.1E-03	5.7E-03	1.0E-02	0.5
5-Methoxyindoleacetic acid or Indole-3-lactic acid	204.067	8.61	4.5E-04	2.5E-04	1.2E-03	0.4
4-Guanidinobutyric acid	146.093	7.78	3.6E-04	4.4E-04	1.1E-03	0.3
Cystine	241.032	10.35	1.8E-03	1.6E-02	5.8E-03	0.3
Ala-Ser or Thr-Gly	177.089	8.96	6.5E-04	1.1E-02	2.4E-03	0.3
*N*-Acetyllysine, (Ala-Val), (Ile-Gly)or(Leu-Gly)	189.124	9.20	3.5E-03	4.4E-02	1.3E-02	0.3
(Asn, Ile), (Asn, Leu) or Val-Gln	246.147	9.84	4.6E-04	6.9E-03	2.6E-03	0.2
(Leu, Ile)	245.188	9.97	3.0E-04	8.9E-03	1.9E-03	0.2
Ile-Gln or Leu-Gln	260.162	10.01	1.6E-04	5.7E-03	1.1E-03	0.14
Pipecolic acid	130.087	9.65	7.3E-04	8.2E-04	5.4E-03	0.13
Ile-Ala or Leu-Ala	203.140	9.37	3.1E-04	9.5E-03	2.5E-03	0.12
Ile-Val	231.171	9.79	1.0E-03	1.5E-02	8.7E-03	0.12
(Ile-Thr) or (Thr-Leu)	233.151	9.80	5.5E-04	1.6E-02	5.2E-03	0.11
Asp-Ile or Asp-Leu	247.130	10.09	8.4E-05	1.0E-02	1.4E-03	0.06
Cytidine	244.094	9.10	7.5E-05	5.5E-03	1.7E-03	0.05
Adenine	136.063	7.17	3.2E-04	1.7E-03	8.0E-03	0.04
Ethylacetimidate	88.075	6.67	3.8E-04	3.3E-04	1.1E-02	0.04
Spermidine	146.166	4.27	ND	2.8E-05	1.2E-03	<1

a Ratio of both relative peak areas, ND indicates not detected.

Kojic acid, produced as a secondary metabolite by the koji mold *Aspergillus oryzae* [19], was detected in Honcho that was made with low water/materials ratio, however, not detected in Sakamoto made using traditional methods. Kojic acid is widely used in the cosmetics industry [19]. Honcho contained more ergothioneine than Sakamoto, although the content was not very high. Ergothioneine, a strong antioxidant [20], is produced by *A. oryzae* [21] and plays a role in human health and disease by providing protection against oxidative stress [22,23].

The mevalonic acid concentration in Honcho was 11 times higher than that in Sakamoto. This substance is produced by koji mold and is known as an essential growth factor for highly ethanol-tolerant lactobacilli, which cause sake spoilage, also known as true hiochi bacteria [24,25]. This suggests a relationship between lactic fermentation in Honcho and sake spoilage, which corresponds to the ancient concept that vinegar originates from spoiled liquor [8]. The dyphylline and 5-aminovaleric acid contents in Honcho were 9.6 times and 33 times higher than those in Sakamoto, respectively. Dyphylline is known for its strong bitter taste (PubChem CID: 3182), which is expected to affect the taste of the final Honcho product.

Most of the 107 peptide candidate peaks were significantly smaller or were not detected in Honcho relative to Sakamoto (data not shown). However, prolyl peptides, Gly-Pro (or Pro-Gly), displayed peaks 3.6 times higher in Honcho than in Sakamoto. Peptides with a prolyl residue at the second position from the N-terminus (X-prolyl peptides) are resistant to many peptidases including aminopeptidases and carboxypeptidases [26]. In soy sauce fermentation, koji mold contains an X-prolyl dipeptidyl peptidase (DppⅣ) that releases Xaa-Pro from X-prolyl peptides and promotes the hydrolysis of protein, producing abundant free amino acids [27]. More koji was used in the Honcho preparation than in the Sakamoto one. Therefore, it was considered that more DppIV acted on X-prolyl peptides to release Xaa-Pro, and more peptidases acted on other peptides, thereby increasing Gly-Pro release and free amino acid production in Honcho compared with Sakamoto.

It took six months to produce Honcho and Sakamoto outdoors, but since Honcho contains more than 10 % ethanol and its pH is below 4 [10], there were no problems with the shelf life of Honcho, similar to Sakamoto. However, since lactic fermentation was completed within the initial two weeks, it may be possible to obtain Honcho with a difference in the minor components in a shorter period.

Since we used spontaneously fermented vinegar samples in this experiment, their minor components might display microflora-dependent variations. However, we observed no major differences in the minor organic acid and trace substance compositions (Table 2, Table 4) between Honcho 1 and 2 with different lactic acid bacterial flora [10]. The main components (e.g., lactic acid and ethanol) of the vinegars we obtained repeatedly over two years showed similar trends [10]. Since this study describes the first metabolome analysis of a lactic acid-fermented rice product, the hereby-obtained data will be valuable for future research efforts.

### 3.4 Volatile compounds

To compare the aromatic components of Honcho, Bankin, and Sakamoto, the volatile substances contained in the mash 199 days after each preparation were analyzed. GC/MS analysis of three lots of Honcho, Bankin, and Sakamoto showed that ethanol and acetic acid presented the largest peaks. Almost identical volatile peaks were detected for Honcho Bankin, and Sakamoto; however, there was a clear difference in their amounts (data not shown). The aromatic components of vinegar are affected by acetic acid fermentation by acetic acid bacteria [28]. Acetaldehyde, ethyl acetate, 1-propanol, isobutanol, isoamyl alcohol, and acetoin, which are affected by acetic acid fermentation [28], were selected for comparison with Honcho and Sakamoto (Table 5). The ethyl acetate content was remarkably low in Honcho (55.31 ± 14.11 mg/L, *p* < 0.01 with Sakamoto), where acetic acid bacteria did not grow [10]. Since ethyl acetate, produced by the esterase of acetic acid bacteria, is the main aromatic component of vinegar [29], the aroma of Honcho was assumed to be largely different from that of Sakamoto. Acetaldehyde in sake is produced from pyruvic acid by yeast [30]. The amount of acetaldehyde was higher in Honcho (127.66 ± 3.46 mg/L) than that in Sakamoto (57.93 ± 21.56 mg/L). During vinegar production, acetaldehyde produced via ethanol oxidation is further oxidized to acetic acid by bacteria [31]. Presumably, acetaldehyde remained in Honcho where acetic acid fermentation did not occur after vigorous alcoholic fermentation. Acetaldehyde is found in some alcoholic beverages, such as sweet wine and sherry, at concentrations of 90–500 ppm, and its concentration in sake is 15–50 ppm [32]. Acetaldehyde concentrations of 80 ppm or higher in sake contribute to its woody odor [33]. In addition, acetaldehyde has a pleasant fruity aroma at low concentrations, and becomes pungent and irritating at higher concentrations [34]. Thus, acetaldehyde was assumed to contribute to the aroma characteristics of Honcho. Unpleasant odors in rice vinegar have long been attributed to acetoin and diacetyl, which have strong butter/cream/cheese-like odors [35,36]. Acetoin in wine is produced by lactic acid bacteria and yeasts during fermentation [37]. Acetoin production in vinegar can be attributed to acetic acid bacteria [36]. Therefore, although Honcho is produced via lactic acid fermentation, its acetoin content was lower than that in Sakamoto (46.81 ± 8.49 mg/L) because acetic acid fermentation did not occur. There was no significant difference in the amount of isobutyl alcohol between Honcho and Sakamoto; however, Honcho contained higher amounts of n-propyl (61.04 ± 32.50 mg/L) and isoamyl alcohols (10.93 ± 2.89 mg/L). These alcohols are the main aromatic compounds produced by yeast in sake [38,39]. These alcohols are converted into unpleasant odorous substances by acetic acid bacteria during vinegar production [28], however, the alcohols produced by yeast were thought to remain in Honcho, where acetic acid fermentation did not occur. Therefore, these alcohols are assumed to contribute to the aroma characteristics of Honcho.

**Table 5:** Selected volatile compounds*^1^ in Honcho, Bankin and Sakamoto.

Volatile compounds	Content (mg/L) *^2^		
	Honcho 1	Bankin	Sakamoto
Acetaldehyde	127.66 ± 3.46^a^	60.07 ± 23.67^b^	57.93 ± 21.56^b^
Ethyl acetate	55.31 ± 14.11^a^	112.99 ± 23.65^a^	458.48 ± 222.93^a^
n-Propyl alcohol	61.04 ± 32.50^a^	85.85 ± 2.37^a^	3.92 ± 0.76^b^
Isobutyl alcohol	5.85 ± 0.93^a^	130.23 ± 5.33^b^	7.97 ± 1.30^ab^
Isoamyl alcohol	10.93 ± 2.89^a^	17.49 ± 2.15^b^	3.41 ± 0.63^c^
Acetoin	18.58 ± 9.19^a^	41.11 ± 52.17^a^	46.81 ± 8.49^a^

*1 Volatile compounds affected by acetic acid fermentation were selected.

*2 Values are represented as mean ± SD (n = 3). Different superscript indicates significant differences at *p* < 0.05.

In our previous study we detected considerable amounts of ethanol in lactic fermented products [10]. Ethanol also plays a major role as a volatile compound in the differences in aroma between Honcho and Sakamoto.

### 3.5 Taste analysis

**Fig. 1:**
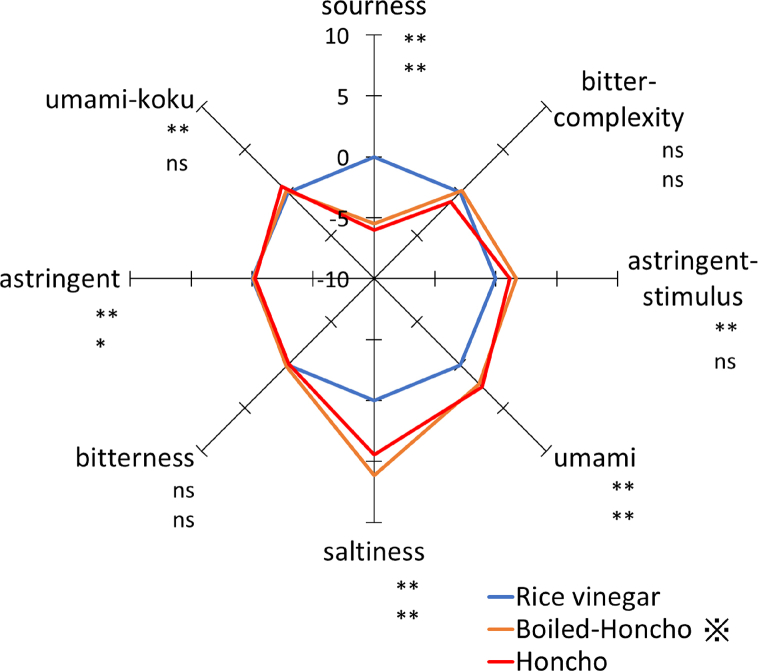
Taste differences between rice vinegar, Honcho 1, and boiled Honcho 1 (interpolation difference value). ※Honcho 1 was boiled to remove ethanol. Significant differences between rice vinegar-Honcho and rice vinegar-boiled Honcho are indicated at the top and bottom, respectively, at *p < 0.05, **p < 0.01, ns: no sense (n= 3).

Using a taste sensor, the tastes of Honcho and boiled Honcho were compared by considering the interpolation difference value from the commercially available rice vinegar. Fig. 1 shows a radar chart of the taste patterns of rice vinegar, Honcho, and boiled Honcho, which was boiled to remove ethanol. The Honcho exhibited a very weak sourness and slightly weak bitter complexity, but a slightly stronger astringent stimulus and stronger umami and salty tastes than rice vinegar. The Honcho also displayed a significantly different taste pattern from rice vinegar. The sourness sensor of this device responds to organic acids, acidic amino acids, and other acids. The lower sourness of the Honcho compared to that of rice vinegar is consistent with the differences in their organic acid content. Astringent stimulus sensor responds to inorganic salts, hydrophobic substances, and bitter amino acids. Bitterness sensor responds to hydrophobic substances, amino acids, and peptides. Umami sensor responds to various amino acids. The stronger umami taste of the Honcho compared with that of rice vinegar is consistent with the higher levels of umami amino acids found in the Honcho, such as glutamic acid and aspartic acid. While there was no significant difference in the taste patterns of boiled Honcho and the Honcho, the former exhibited slightly stronger bitterness and saltiness than the latter. The manufacturer states that the salt taste sensor in this device detects chloride ions, minerals, and organic acid salts, which contribute to the intensity and richness of the taste.

### 3.6 Sensory evaluation of Honcho and Honcho dishes

To investigate the flavor characteristics of Honcho and assess its usefulness as a seasoning, sensory evaluation of Honcho was conducted by skilled chefs.

**Fig. 2:**
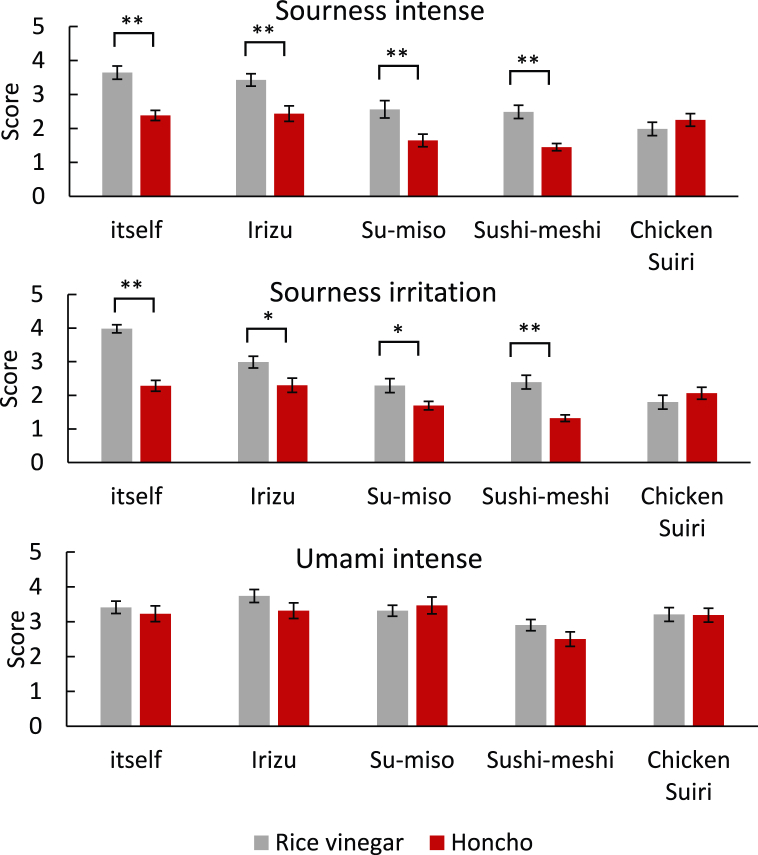
Sensory evaluation of Honcho 1 and dishes with Honcho by 19 skilled chefs. n = 19, *p < 0.05, **p < 0.01.

As shown in Fig. 2 rice vinegar dishes scored significantly higher than the Honcho dishes in terms of sourness intensity and stimulus. This result suggests that Honcho is mildly acidic. In contrast, there were no significant differences in Suiri, where acetic acid was sufficiently volatilized through heating, and the score was almost the same for both items. No significant differences were observed regarding umami, suggesting that Honcho has the same palatability as rice vinegar. We previously reported that vinegar was characteristically used for Suiri in the Edo period, which is rarely observed in modern Japanese cuisine [40]. Suiri is a vinegar-cooking technique that involves boiling, baking, and steaming. Based on this result, Honcho was found to be less sour than the rice vinegar. However, dishes using Honcho showed a stronger sourness in the Suiri heat-cooking method, suggesting the suitability of lactic fermented products for cooking.

### 3.7 Changes in acidity of vinegars during cooking

**Fig. 3:**
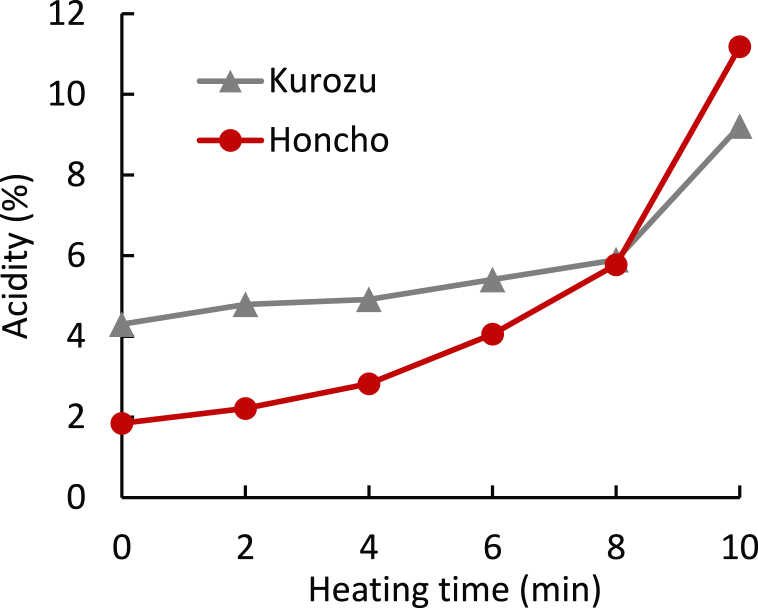
Changes in Honcho and Kurozu acidity during cooking. Kurozu and Honcho 1 (100 mL each) were placed in a 1 L stainless steel beaker and heated using an induction cooker.

Acetic acid, the main component of vinegar, is a volatile acid, whereas the Honcho acidity is derived mainly from lactic acid, which is a non-volatile acid. Therefore, a difference in acidity between the two is expected after cooking. Kurozu and Honcho 1 (100 mL each) were placed in a 1 L stainless steel beaker and heated using an induction cooker. We compared the changes in the acidity of Honcho and Kurozu upon heating. Fig. 3 shows the change in the acidity when 100 mL of each vinegar sample was boiled for 10 min. The acidity of Honcho before boiling was approximately half of that of Kurozu. The acidity of both samples gradually increased with boiling; however, the increase in Honcho was clearly greater. Although acetic acid is a volatile acid, it is less likely to volatilize than water because it has a boiling temperature of 118 °C [41]. It is considered that Kurozu acidity gradually increased because both acetic acid and water gradually volatilized with increasing heating time. In contrast, acidity possibly increased significantly in Honcho because of the rapid volatilization of ethanol (approximately 10 % content), causing non-volatile acids, such as lactic acid, to concentrate. After 8 min of heating, the acidity of Honcho increased almost 2-fold; after 10 min, Honcho was more acidic than Kurozu. Sourness thresholds of acetic acid and lactic acid are 3.6 mg/L and 3.7 mg/L, respectively [42]. It was considered that because lactic acid concentration in Honcho was higher than the acetic acid concentration in Kurozu after concentrated by boiling.

Our previous report showed that Japanese cookbooks from the mid-17th to 19th centuries contained a relatively large number of cooked dishes made using vinegar, which is not often seen in modern Japanese cuisine [40]. The sensory evaluation in this study was based on modern vinegar cooking, which is not optimal for Honcho. If the amount used was based on Honcho's acidity, and twice the volume of Honcho or Honcho boiled to concentrate to a half volume was used instead of modern vinegar, it would be possible to obtain a better evaluation of cooked dishes. In addition, the sour taste of Honcho, which has a different flavor, would have seemed strange to the panelists, who are familiar with modern vinegar because modern vinegar has an irritating odor, whereas Honcho has no irritating odor and has an umami taste.

The Edo cookbooks frequently describe Namasu as the most popular vinegar dish, accounting for 44 % of all the vinegar dishes described. Namasu is a dish made by cutting raw fish meat into thin pieces and soaking them in vinegar [40]. Vinegar is thought to have contributed to the preservation of Namasu; however, the lactic acid in Honcho has a weaker antibacterial effect than acetic acid [43]. Despite this fact, Honcho still retains a small percentage of alcohol even after heat sterilization, which could explain its preservative properties. In the Suiri cooking method, raw materials, such as fish and shellfish, were simmered in vinegar, fried with vinegar, or poured with hot vinegar [40]. The characteristics of Honcho, such as its richness in non-volatile acids and amino acids, make it particularly well-suited for its use in Suiri.

This result supports the hypothesis that this lactic fermented product was used to acid-season Japanese cuisine until it was replaced with modern vinegar at the end of the Edo period.

## 4 Conclusion

Chemical analysis was performed on the flavor components of Honcho, a lactic fermented product reproduced from the Edo period book *Honchoshokkan*. Honcho differs from modern rice vinegar in terms of sourness, taste, and aroma components. Although Honcho has a lower acidity than modern rice vinegar, its free glutamic acid content is much higher. Therefore, unlike modern vinegar, it may have been a seasoning product with an umami taste similar to that of soy sauce. Non-volatile acids in Honcho were concentrated when heated, suggesting that Honcho was used as a sour seasoning in cooked dishes, such as Suiri, which often appeared in the Edo period cookbooks. Sensory evaluation confirmed that the Honcho seasoning makes dishes palatable by adding umami and non-stimulus sourness. Vinegar dishes up to the Edo period may have been seasoned differently from what we had previously thought. For people at that time who could not distinguish between acetic and lactic fermentation, it would not have been a failure for the sour-fermented product to be obtained by lactic fermentation during the production of rice vinegar, as it could be used for cooking as a seasoning with sourness and umami. The Honcho flavor characteristics presented in this study support our hypothesis that lactic fermented products may have been produced as vinegar and used to add sourness and umami to Japanese dishes until they were replaced with modern vinegar in the 19th century. As the actual product does not exist, it is impossible to verify whether the lactic fermented product was actually produced. The findings of this research will greatly contribute to replicate the cooking and cuisine during the Edo period and the development of new seasonings that reproduce the traditional Japanese taste. Detailed laboratory-scale validation studies are currently underway.

## Data availability statement

Data will be made available on request.

## CRediT authorship contribution statement

**Naoyuki Yanagihara:** Writing – original draft, Visualization, Validation, Methodology, Investigation, Formal analysis, Data curation. **Maeda Mayumi:** Writing – original draft, Investigation, Data curation. **Jun Yoshikawa:** Writing – review & editing. **Sayuri Akuzawa:** Writing – original draft, Methodology. **Akira Fujii:** Writing – review & editing. **Masanobu Nagano:** Writing – review & editing. **Yukimichi Koizumi:** Writing – review & editing. **Kenji Maehashi:** Writing – original draft, Supervision, Project administration, Methodology, Conceptualization.

## Declaration of competing interest

The authors declare that they have no known competing financial interests or personal relationships that could have appeared to influence the work reported in this paper.
