# Supplementary material for: Flavor assessment of a lactic fermented vinegar described in Japanese books from the Edo period (1603–1867)
Source: Heliyon. 2024 Jun 4;10(11):e32344. doi: 10.1016/j.heliyon.2024.e32344 (PMC11219324; doi:10.1016/j.heliyon.2024.e32344)
Supplement: Multimedia component 2 [file mmc2.pptx]

## Slide 1
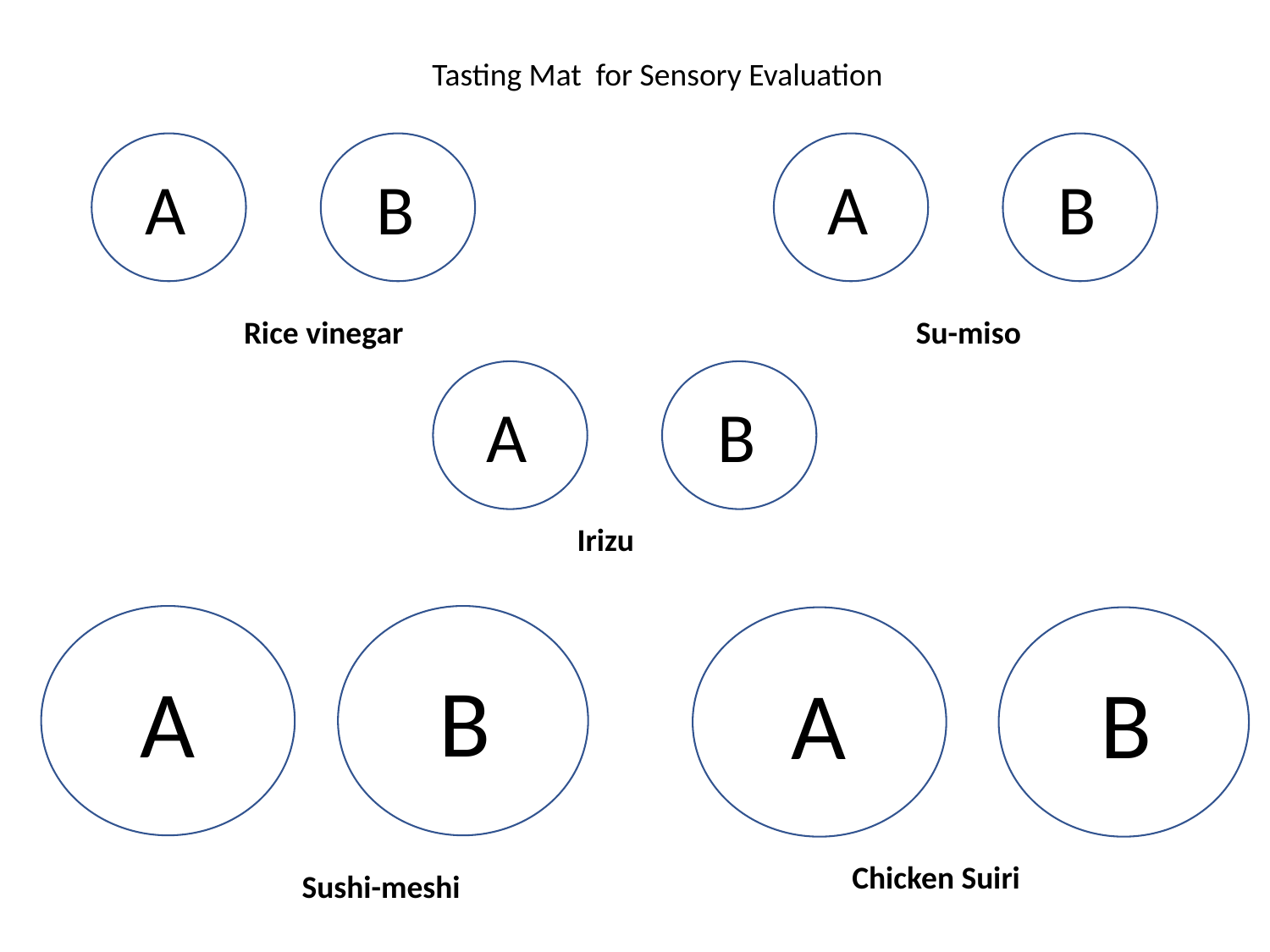

Tasting Mat for Sensory Evaluation
A
B
A
B
Rice vinegar
Su-miso
A
B
Irizu
B
A
B
A
Chicken Suiri
Sushi-meshi
