# Supplementary material for: Flavor assessment of a lactic fermented vinegar described in Japanese books from the Edo period (1603–1867)
Source: Heliyon. 2024 Jun 4;10(11):e32344. doi: 10.1016/j.heliyon.2024.e32344 (PMC11219324; doi:10.1016/j.heliyon.2024.e32344)
Supplement: Multimedia component 3 [file mmc3.pdf]

令和３年　９月　27 日

東京農業大学
学長　　江口　文陽　殿

人を対象とする実験・調査等に関する研究計画書

下記のとおり人を対象とする実験・調査等に関する研究を行いたく，審査をお願いいたします。

申請者(研究代表責任者)　所属・職名　　醸造科学科・教授

※研究代表責任者として記載可能な職名は、教員、非常勤講師、客員教授、客員研究員、博士研究員、学術研究員です

氏名　　前橋　健二　　印

所属長氏名　　醸造科学科長　前橋　健二　　印

研究題目		江戸期書物記載の製法による米酢の品質について			
研究期間		令和３年 10 月１日 ～ 令和 ４年 ９月 30 日			
		氏名	所属	職名	担当内容
研究組織	構　成　員<br>※学内者で記載可能な職名は次のとおり。教員、非常勤講師、客員教授、客員研究員、博士研究員、学術研究員、特別研究員、リサーチ・アシスタント、大学院生、大学院所属の研究生	前橋健二<br>柳原尚之	醸造科学科<br>醸造学専攻	教授<br>博士後期課程３年	同意書取得、統計解析<br>サンプル調製、官能評価、データ管理
研究内容(目的、意義、方法、期待される成果)		江戸期書物に記載されている米酢製造の再現実験によって得られた米酢について、官能的特性を明らかにすることによって、江戸期の酢の特徴を明らかにする。			
実験場所		対象者の職場である都内レストランおよび受託業者が指定する場所			
対象者およびその人数		和食、洋食、中華の料理人約 20 名及び一般成人約 10 名			
対象者の負担および人権擁護・個人情報保護のための配慮や安全管理の方策		対象者の情報は官能評価実行者のみが管理し本人の同意なしに公開しない。実験に用いる酢は、酢メーカー管理下で製造されたものとし、酢料理の作製は、料理としての常識の範囲の酢使用量で行う。実験中または後に本実験に起因すると思われる体調異常を万が一でも感じることがあれば直ちに実験を中止し申告させる。			
倫理委員会判断		(承認)　条件付承認　変更勧告<br>不承認　審査対象外			
決裁		令和 3年10月4日<br>委員長　上岡洋晴　印<br>2114			

Ver.20210401
